# Developmentally regulated expression of integrin alpha-6 distinguishes neural crest derivatives in the skin

**DOI:** 10.3389/fcell.2023.1140554

**Published:** 2023-05-15

**Authors:** Shize Ma, Xiu Li, Rui Cao, Guoqin Zhan, Xin Fu, Ran Xiao, Zhigang Yang

**Affiliations:** ^1^ Plastic Surgery Hospital, Chinese Academy of Medical Sciences and Peking Union Medical College, Beijing, China; ^2^ Key Laboratory of External Tissue and Organ Regeneration, Chinese Academy of Medical Sciences, Beijing, China

**Keywords:** neural crest, skin, melanocyte, Schwann cell, fibroblast, integrin α6, Wnt1-Cre

## Abstract

Neural crest-derived cells play essential roles in skin function and homeostasis. However, how they interact with environmental cues and differentiate into functional skin cells remains unclear. Using a combination of single-cell data analysis, neural crest lineage tracing, and flow cytometry, we found that the expression of integrin α6 (ITGA6) in neural crest and its derivatives was developmentally regulated and that ITGA6 could serve as a functional surface marker for distinguishing neural crest derivatives in the skin. Based on the expression of ITGA6, Wnt1-Cre lineage neural crest derivatives in the skin could be categorized into three subpopulations, namely, ITGA6^bright^, ITGA6^dim^, and ITGA6^neg^, which were found to be Schwann cells, melanocytes, and fibroblasts, respectively. We further analyzed the signature genes and transcription factors that specifically enriched in each cell subpopulation, as well as the ligand or receptor molecules, mediating the potential interaction with other cells of the skin. Additionally, we found that *Hmx1* and *Lhx8* are specifically expressed in neural crest-derived fibroblasts, while *Zic1* and homeobox family genes are expressed in mesoderm-derived fibroblasts, indicating the distinct development pathways of fibroblasts of different origins. Our study provides insights into the regulatory landscape of neural crest cell development and identifies potential markers that facilitate the isolation of different neural crest derivatives in the skin.

## 1 Introduction

The skin is a complex organ system composed of multiple types of cells that function collaboratively and complement each other. The different types of skin cells have different embryonic origins. For example, the epidermis is ectodermal in origin, the fibroblasts in ventral and dorsal skin are derived from the lateral plate mesoderm and the dermomyotome, respectively, and those in the head and face skin originate in the neural crest (Driskell and Watt, 2015). The neural crest is a population of multipotent cells that forms during vertebrate embryogenesis at the border between the neural plate and the prospective epidermis. Neural crest cells delaminate through an epithelial-to-mesenchymal transition and migrate extensively throughout the embryo. They eventually settle and differentiate into multiple cell types (Bronner, 2012), including neurons and glial cells of the peripheral nervous system, pigment cells, and craniofacial skin mesenchymal cells (Nitzan et al., 2013; Thulabandu et al., 2018). Abnormal neural crest development can lead to defective skin structure and function, including craniofacial skin deformities, pigmentation disorders, and neurocutaneous syndromes (Takahashi et al., 2013). Accordingly, understanding the regulatory landscape of different neural crest cell derivatives in the skin is likely to offer a novel perspective on neural crest development and related skin disorders.

The integrins are a large family of cell adhesion receptors, each of which is composed of different combinations of alpha and beta subunits that can assemble into 24 distinct heterodimers (Takada et al., 2007). These heterodimer combinations have unique affinities for extracellular matrix (ECM) components, such as collagens, laminins, and fibronectin. As transmembrane receptors, integrins transmit both “inside-out” and “outside-in” signals by linking the ECM and the cytoskeleton (Shen B. et al., 2012). The activation of integrins upon ligand binding triggers signal transduction mechanisms involved in cell proliferation, differentiation, motility, polarity, and survival/apoptosis (Streuli, 2009; Wrighton et al., 2014). Integrin α6 (ITGA6), also known as CD49f, can form heterodimers with either integrin β1 or integrin β4 and function as a receptor for laminins. ITGA6 has been identified as a robust marker for stem cells of different sources, including epithelial, neural, hematopoietic, germline, and mesenchymal stem cells, and regulates their biological behaviors through cross-talk with environmental cues (Krebsbach and Villa-Diaz, 2017; Xu et al., 2019; Yang et al., 2020; Zha et al., 2021). The expression of ITGA6 has also been found to negatively affect the migration of neural crest cells and play a role in their subsequent differentiation (Strachan and Condic, 2003; Kim et al., 2014; Corallo et al., 2020; Thomas et al., 2022).

Here, we employed a Wnt1-Cre2 mouse line, which is widely used in neural crest lineage tracing (Lewis et al., 2013), to study the distribution and development of neural crest in the skin in different body regions. Interestingly, we found that neural crest derivatives in the skin exist as three distinct cell subpopulations based on the expression level of ITGA6, namely, ITGA6^neg^, ITGA6^dim^, and ITGA6^bright^. While all three subpopulations could be clearly observed in the facial skin, ITGA6^neg^ cells could barely be detected in the trunk skin. An *in vitro* differentiation assay showed that ITGA6^bright^ cells preferentially differentiated into Schwann cells; ITGA6^dim^ displayed typical melanocyte morphology concomitant with high expression levels of *Mitf*, *Dct*, *Kit*, and *Mlana*; and ITGA6^neg^ cells could effectively be induced to adipocytes. Transcriptomic analysis confirmed the identities of ITGA6^bright^, ITGA6^dim^, and ITGA6^neg^ cells as Schwann cells, melanocytes, and fibroblasts, respectively, and further revealed the specific transcription factors, ligands, and receptors that regulate the differentiation of neural crest cells in the skin. Additionally, by comparing ITGA6^neg^ cells with the non-neural crest-derived fibroblasts, we identified *Hmx1* and *Lhx8* as potential regulators of neural crest-derived fibroblasts and *Zic1* as one of the somite-derived fibroblasts. Our study identified ITGA6 as a developmentally regulated neural crest marker, which facilitates the identification and isolation of neural crest derivatives in the skin and provides insights into the regulatory landscape of neural crest cell development and maintenance.

## 2 Materials and methods

### 2.1 Single-cell RNA sequencing data acquisition and analysis

The single-cell expression data from GSE201257 were downloaded from the Gene Expression Omnibus (http://www.ncbi.nlm.nih.gov/geo/) database. The R package Seurat was used for normalization, principle component analysis, variable gene finding, clustering analysis, and t-distributed stochastic nearest neighbor embedding (Hao et al., 2021). Pseudotime trajectory analysis was performed with the R package Monocle (version 2.18.0) with default settings unless otherwise specified (Qiu et al., 2017). Genes used for pseudotime ordering were taken from the differentially expressed genes identified by function VariableFeatures. The DDRTree method was utilized for dimension reduction and cell ordering along with the pseudotime trajectory. CytoTRACE, an algorithm leveraged that transcriptional diversity decreases during differentiation (Gulati et al., 2020), was applied to predict differentiation states. A protein–protein interaction (PPI) network was constructed using STRING (version 11.5) (Szklarczyk et al., 2023) and visualized using Cytoscape (Shannon et al., 2003).

### 2.2 Mouse models

Wnt1-Cre2 (JAX stock #022501) (Lewis et al., 2013) were crossed to the reporter strain Rosa26-mTmG mice (a kind gift from Dr. Dahai Zhu) to generate Wnt1-Cre2; Rosa26-mTmG mice. All mice were housed on a 12-h light–dark cycle in a controlled climate, and all animal experiments were conducted in accordance with the guidelines and prior approval of the Animal Care and Use Committee at the Plastic Surgery Hospital, PUMC.

### 2.3 Cell isolation and flow cytometry

The trunk and vagal neural tube of E9.5 Wnt1-Cre2; Rosa26-mTmG embryo were isolated and dissociated to single cells using 0.1% collagenase as previously reported (Pfaltzgraff et al., 2012). The craniofacial and trunk skin from P0 newborn mice were dissected and incubated in 0.25% Dispase II overnight. The epidermal sheets were peeled off and dissociated by 0.25% trypsin. The dermal tissues were cut into 2–3^m3^ pieces, digested with 0.1% collagenase for 2 h at 37°C, filtered through a 40-μm cell strainer (Falcon, BD Biosciences, SanDiego, CA), and then washed in PBS and stained with antibodies for flow cytometry analysis or sorting. For each staining, 2 μL of antibody was added per million cells in 100 μL volume, respectively. The antibodies used were ITGA6- PE-Vio770 (Miltenyi, 130-107-779), MCAM-APC (BioLegend, 134711), KIT-APC (BioLegend, 105811), ITGA7-APC (Invitrogen, MA5-23555), ITGA1-Alexa Fluor 647 (BD Biosciences, 562113), GPNMB-APC (eBioscience, 50-5708-82), and CDH1 (BioLegend, 147309). 7-AAD Viability Staining Solution (eBioscience, 00-6993-50) was used to eliminate dead cells. Cell analysis and sorting were performed with a FACS Aria II (BD Biosciences, San Jose, CA), and data were analyzed using FlowJo software (Tree Star, Inc., Ashland, OR).

### 2.4 Cell culture and differentiating induction

FACS-isolated ITGA6^neg^, ITGA6^dim^, and ITGA6^bright^ cells from craniofacial and trunk skin were cultured in DMEM: F12 medium (3:1) containing 20 ng/mL FGF2, 10 ng/mL EGF, and 2% B27 supplement. For Schwann cell differentiation, the cells were cultured in the medium composed of DMEM: F12 and 5 μm forskolin, 50 ng/mL heregulin-1β, 2% N2 supplement, and 1% FBS for 2 weeks. Melanocytes (Kim et al., 2014) were differentiated by maintenance in Melanocyte Cell Basal Medium-4 containing endothelin-3, MGM-4 Bullet Kit, and MBM-4 plus SingleQuots of Growth Supplements for 1 week. For *in vitro* differentiation of adipocytes, FACS-sorted dermal cells were directly plated on 6-well culture plates with DMEM containing 10% FBS. After reaching confluence, the cells were cultured in specific medium consisting of DMEM with 10% (v/v) FBS, 1 μM dexamethasone, 200 μM indomethacin, 0.5 mM 3-isobutyl-1-methylxanthine, and 10 μM insulin for 7 days.

### 2.5 Immunocytochemistry

The skin tissues from newborn Wnt1-Cre2; Rosa26-mTmG mice (P0) were fixed with 4% paraformaldehyde for 24 h at 4°C, dehydrated with 10% sucrose overnight before cryosectioning (10 μm). After blocking, anti-GFP and anti-Krt14 primary antibodies (Abcam, ab181595, 1:100), and Alexa Fluor 488 couple anti-Chicken IgY (Invitrogen, A-11039, 1:500) and Alexa Fluor 594 coupled anti-rabbit IgG (Invitrogen, A-11037, 1:500) were sequentially incubated with the sections. Nuclei were stained with Hoechst dye (1:1,000, Sigma-Aldrich, St. Louis, MO). For staining of differentiated cells, antibodies for GFP, P75 (Abcam, ab52987, 1:100), tubulin-β (Abcam, ab18207, 1:100), and PMEL (Abcam, ab137078, 1:50) were applied. Fluorescent images were acquired using an immunofluorescence microscope (Leica, Wetzlar, Germany).

### 2.6 Quantitative RT-PCR

Total RNA was extracted with TRIzol reagent (Thermo Fisher, United States), and 1 μg RNA was reverse-transcribed into cDNA using oligo dT primer and M-MLV reverse transcriptase (Promega, United States). Quantitative RT-PCR was used using a Fast SYBR Green Master Kit and Light Cycler 480 system (Roche, Switzerland) according to the manufacturer’s instructions. The expression level of each target gene was normalized to GAPDH and measured by the comparative CT method (ΔΔCT). Primer sequences used are listed in Table 1.

**TABLE 1:** Primer sequences.

Gene	Forward	Reverse
*Mbp*	TGT​GAG​AGT​CCA​GAG​TGG​GG	AAG​TCC​CCG​TTT​CCT​GTT​GG
*Mpz*	GGA​AGG​ATG​GCT​CCA​TTG​TCA	CTC​CCA​ACA​CCA​CCC​CAT​AC
*Ngfr*	CCG​CTG​ACA​ACC​TCA​TTC​CT	TGT​CGC​TGT​GCA​GTT​TCT​CT
*Sox10*	TTT​CTT​ACA​TGG​GGC​CCT​CC	GCC​CCT​CTA​AGG​TCG​GGA​TA
*Dct*	CCT​GAA​TGG​GAC​CAA​TGC​CT	AGG​CAT​CTG​TGG​AAG​GGT​TG
*Kit*	CTC​ACA​TAG​CAG​GGA​GCA​CA	GCT​GTT​ACG​TCT​TGG​GTC​CT
*Mitf*	GAC​TAT​GGC​CAA​GGC​AGA​GC	TTA​GGA​GGA​AGG​TGC​CTG​GA
*Mlana*	GCT​TAT​CGG​CTG​CTG​GTA​CT	CTT​CTC​ATA​GGC​AGG​CGG​AG
*Adipoq*	GAG​GTG​GGA​GAC​CAA​GTC​TG	GCT​GAA​AGT​GTG​TCG​ACT​GT
*Pparg*	AAG​GTG​CTC​CAG​AAG​ATG​ACA​G	TTG​TCA​GCG​GGT​GGG​ACT​T
*Fabp4*	TTG​GTC​ACC​ATC​CGG​TCA​GA	GGT​CGA​CTT​TCC​ATC​CCA​CT
*Plin1*	ATG​CCC​TGA​AGG​GTG​TTA​CG	CCT​CGG​TTT​TGT​CGT​CCA​GG
*Gapdh*	CTA​CCC​CCA​ATG​TGT​CCG​TC	GCC​GTA​TTC​ATT​GTC​ATA​CCA​GG

### 2.7 RNA-seq and data analyses

Total RNA was extracted with TRIzol reagent, and mRNA was purified from total RNA using poly-T oligo-attached magnetic beads. Sequencing libraries were generated using NEBNext^®^ UltraTM RNA Library Prep Kit for Illumina^®^ (NEB, United States) following the manufacturer’s recommendations, and index codes were added to attribute sequences to each sample. After clustering of the index-coded samples by cBot Cluster Generation System using TruSeq PE Cluster Kit v3-cBot-HS (Illumina), and the library preparations were sequenced on an Illumina NovaSeq platform and 150 bp paired-end reads were generated.

After quality control, the clean reads were mapped to the reference genome (mm10) using HISAT2. featureCounts v1.5.0-p3 was used to count the read numbers mapped to each gene. Then, the FPKM of each gene was calculated based on the length of the gene and read counts mapped to this gene. Genes with an adjusted *p*-value <0.05 found using DESeq2 were assigned as differentially expressed. Functional annotation and enrichment analyses were performed using the DAVID online tool, and the results were visualized using the R package “GOplot.” Hierarchical clustering analyses for the samples were performed with an FPKM matrix of selected DEGs, and the heatmap was subsequently generated by pheatmap. For transcriptional regulatory network analysis, transcription factors that may regulate the differentially expressed genes were predicted using ChEA3 (Keenan et al., 2019) and visualized using Cytoscape software (Shannon et al., 2003). The raw sequence data have been deposited in the National Genomics Data Center under BioProject accession number: PRJCA014278.

### 2.8 Statistical analysis

Data were analyzed using Prism 5.0 (GraphPad Software, Inc., United States of America). All values are expressed as the mean ± SEM. Unless otherwise indicated, the differences between the groups were assessed using Student’s t-test. The results were considered statistically significant at *p* < 0.05.

## 3 Results

### 3.1 Single-cell transcriptomic analysis revealed a potential role for adhesion molecules in neural crest development

**FIGURE 1:**
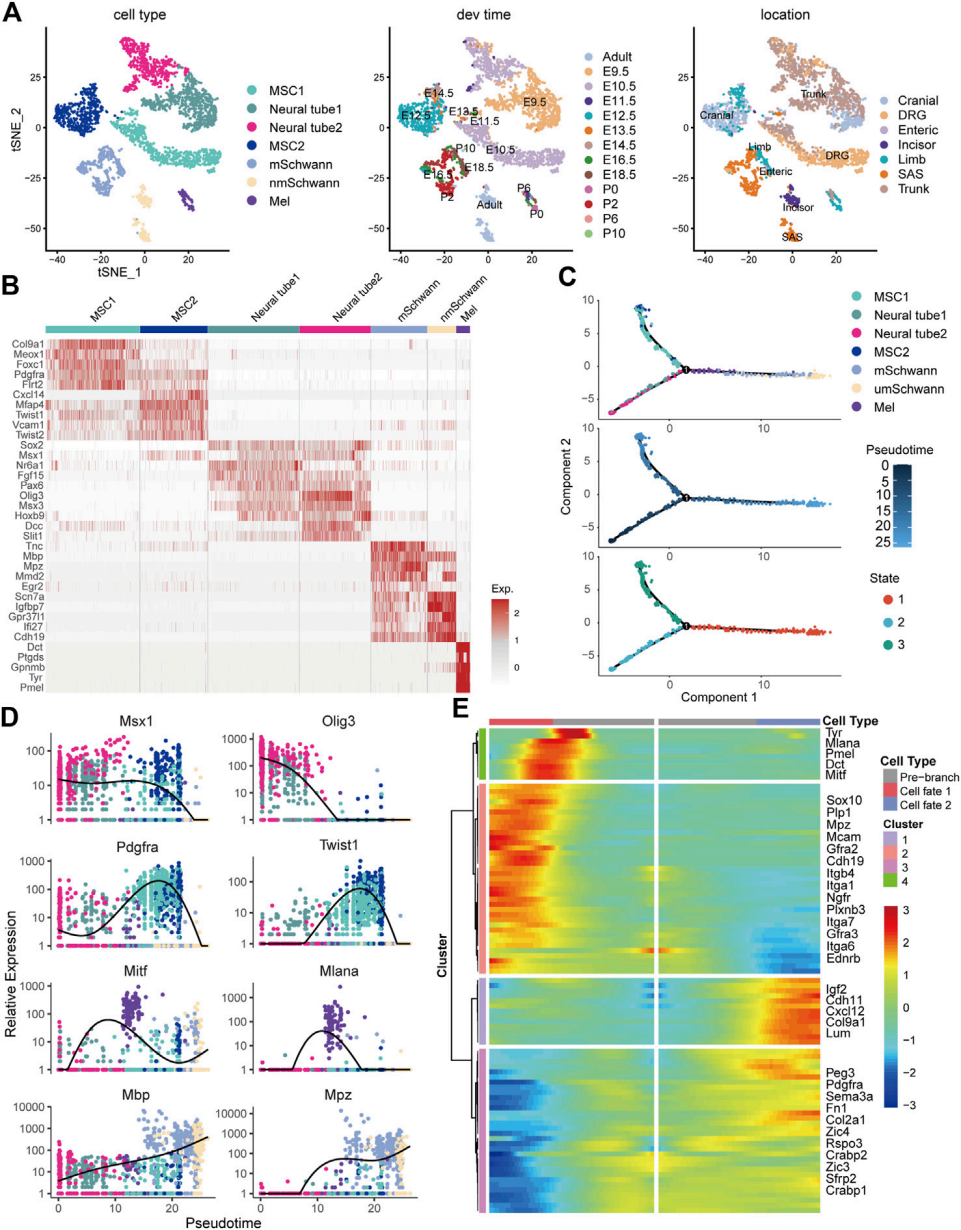
Single-cell transcriptomic analysis of neural crest cells. **(A)** Seven cellular clusters were revealed by unbiased cluster analysis and color-coded projections of developmental time and tissue of origin. **(B)** Heatmap of marker genes in each cluster. **(C)** Ordering of cells along the pseudotime trajectory and distinct states of cells identified by pseudotime analysis. **(D)** Relative expression levels of the markers for each cluster along the pseudotime trajectory. **(E)** Pseudotime heatmap of the significantly changed genes in the branch point discovered by the BEAM function from Monocle 2. MSCs, mesenchymal stem cells; mSchwann, myelinating Schwann cells; nmSchwann, non-myelinating Schwann cells; Mel, melanocytes; DRG, dorsal root ganglia; SAS, sympathoadrenal system.

To understand the molecular mechanisms involved in the regulation of neural crest cell development in the skin, we re-analyzed the single-cell RNA sequencing data from the GSE201257 dataset (Kastriti et al., 2022), which included neural crest cells and their progenies at different anatomical locations from E9.5 to adult, and re-clustered neural tube cells, myelinating and non-myelinating Schwann cells, melanocytes, and mesenchymal cells based on the expression of specific markers (Tasdemir-Yilmaz et al., 2021; Kastriti et al., 2022) (Figures 1A, B). The different types of neural crest-derived cells are presented at the expected developmental stage and location (Figure 1A). For example, neural tube cells were mainly found in the trunk at E9.5 and E10.5, while mesenchymal cells were primarily found in the cranium and dorsal root ganglia (DRG) from E10.5 to E14.5. Pseudotime analysis indicated that the seven clusters were ordered along the pseudotime trajectory, and three different cell states with one branching point were identified (Figure 1C). The neural tube cells were found at the beginning of the trajectory (State 2), mesenchymal cells at the one end of the trajectory (State 3), and melanocytes and Schwann cells at the other end (State 1) (Figure 1C), as evidenced by the pseudotime change in the expression of marker genes in the different clusters (neural tube markers: *Msx1* and *Olig3*; mesenchymal cell markers: *Pdgfra* and *Twist1*; melanocyte markers: *Mitf* and *Mlana*; and Schwann cell markers: *Mbp* and *Mpz*) (Figure 1D). We also applied CytoTRACE to predict differentiation states for each cluster of the dataset and found a progressively decreasing differentiation pattern from neural tube cells to Schwann cells (Supplementary Figures S1A, S1B), which is consistent with the pseudotime analysis result by Monocle. To further demonstrate the regulation of neural crest cell development, we took advantage of branch point 1 analysis and identified specific gene clusters that regulate cell fate determination toward melanocyte and Schwann cell lineages (e.g., *Mitf*, *Sox10*, *Tyr*, *Dct*, and *Ngfr*) or a mesenchymal cell lineage (e.g., *Pdgfra*, *Lum*, and *Sfrp2*) (Figure 1E). Interestingly, we found that the expression of many genes encoding adhesion molecules changed dynamically during differentiation, including *Itga1*, *Itga6*, *Itga7*, *Itgb4*, *Mcam*, *Ednrb*, *Cdh19*, and *Gfra2* (Figure 1E). As integrins are of our interest, we further checked their expression and found a distinctive cell cluster specific pattern. For example, *Itga4*, a well-recognized player in neural crest development, showed the highest expression level in melanocytes, whereas *Itga6* was most abundantly expressed in myelinating Schwann cells. *Itga6* could discriminate all cell clusters due to their hierarchical expressions in different cell populations and was identified as a hub gene with the highest degree score among extracellular matrix molecules and their receptors, suggesting a regulatory role in neural crest development (Supplementary Figures S1C, S1D).

### 3.2 Neural crest derivatives in the skin exist as three subpopulations

**FIGURE 2:**
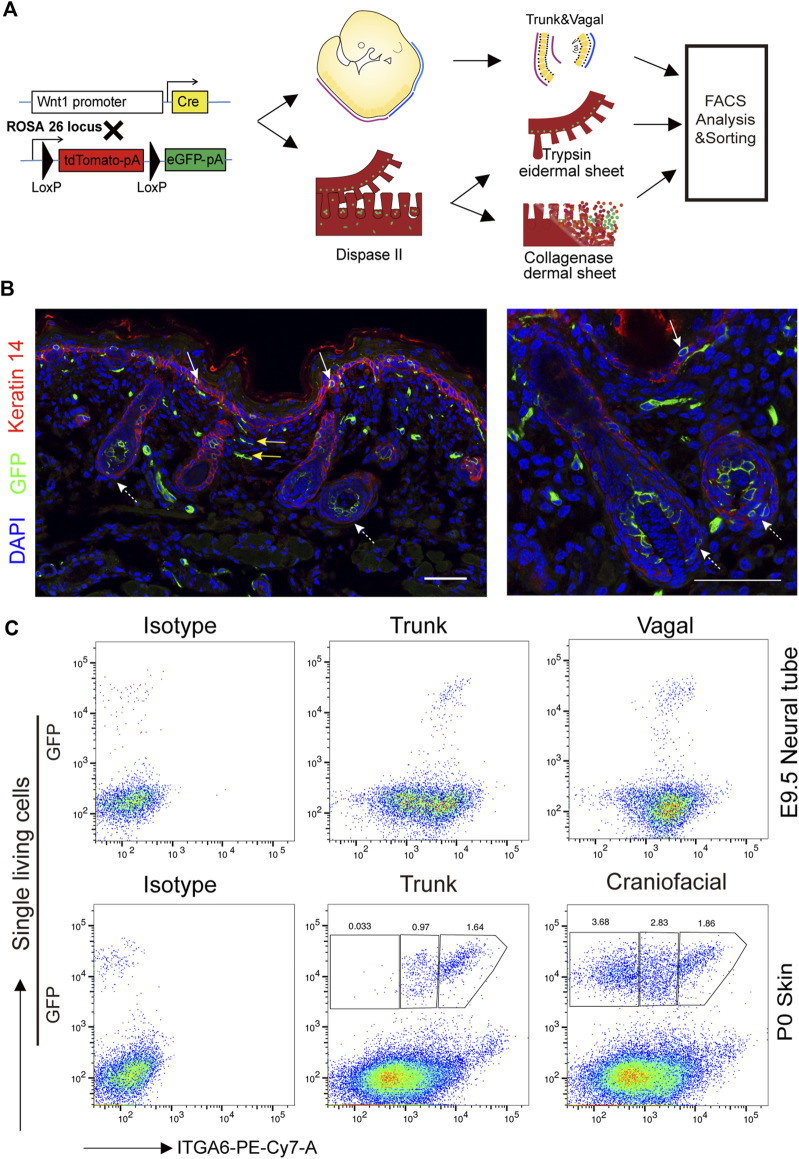
Expression of ITGA6 on the neural crest cells and their derivatives in the skin. **(A)** Schematic illustration of the isolation of neural crest cells and their derivatives in the skin. **(B)** Immunofluorescence staining for keratin 14 and GFP+ cells in the skin of P0 Wnt1-Cre2; mTmG mice; white arrows, GFP+ cells within the epidermis; yellow arrows, GFP+ cells within the dermis; dotted arrows, GFP+ cells within the hair follicle; scale bar = 50 μm. **(C)** Flow cytometric analysis of ITGA6 expression on the trunk and vagal neural tube of E9.5 and the skin of P0 mice.

To comprehensively characterize neural crest cells and their derivatives in the skin, we crossed Wnt1-Cre2 mice, which are widely used in neural crest lineage tracing, to mice harboring a Rosa26-mTmG reporter allele, which labels neural crest cells and their progeny with EGFP (Figure 2A). Immunofluorescence staining showed that GFP+ cells were distributed in the epidermal basal layer, the epidermal/dermal sheet interface, the dermis tissue, and within hair follicles (Figure 2B). Additionally, undifferentiated GFP+ neural crest cells isolated from the trunk and vagal neural tube of E9.5 embryos showed strong staining for ITGA6, as determined by flow cytometry (Figure 2C). In the craniofacial skin of newborn mice, based on the expression level of ITGA6, GFP+ cells could be partitioned into three subpopulations, namely, ITGA6^neg^, ITGA6^dim^, and ITGA6^bright^ cells, representing 3.68, 2.83, and 1.86% of all the skin cells, respectively. Interestingly, in the trunk skin, while both ITGA6^dim^ and ITGA6^bright^ cells could clearly be observed, accounting for 0.97 and 1.64% of the total number of skin cells, respectively, ITGA6^neg^ cells were barely detected and constituted only a very small proportion of the total cells (0.033%) (Figure 2C). The aforementioned results suggested that the expression of ITGA6 in neural crest cells is developmentally regulated and that, following delamination from their original territory between the dorsal neural tube and overlying ectoderm, the neural crest cells migrate to the skin and differentiate into ITGA6^neg^, ITGA6^dim^, and ITGA6^bright^ subpopulations.

### 3.3 Neural crest-derived subpopulations displayed distinct differentiation potentials

**FIGURE 3:**
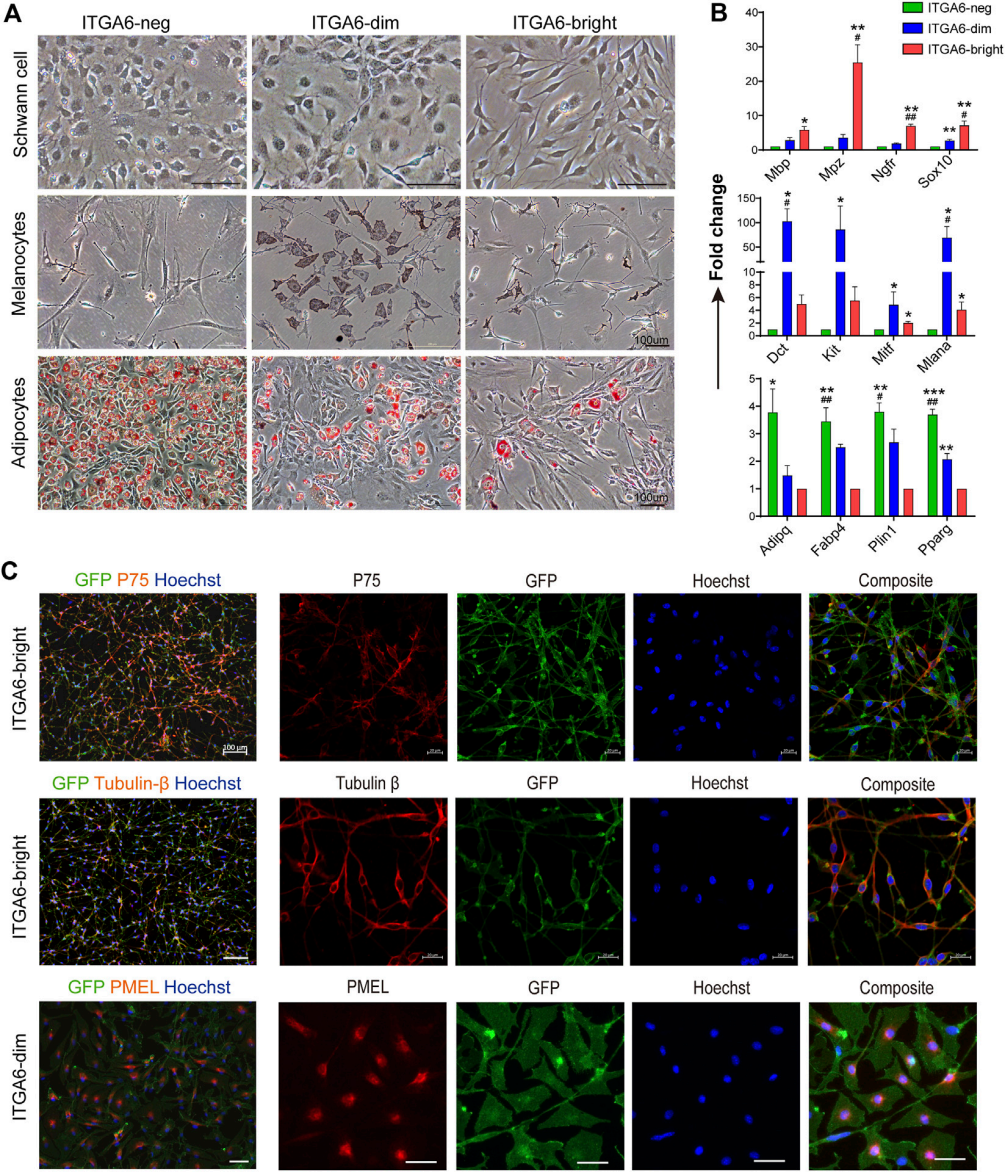
Neural crest-derived subpopulations displayed distinct differentiation potentials. **(A)** Phase-contrast images showing distinct differentiation potentials of ITGA6^neg^, ITGA6^dim^, and ITGA6^bright^ subpopulations toward Schwann cell (top), melanocyte (middle), and adipocyte lineages, respectively, as indicated by Oil red O staining (bottom); scale bar = 100 μm. **(B)** qRT-PCR analysis of the marker genes for Schwann cells (*Mbp*, *Mpz*, *Ngfr*, and *Sox10*), melanocytes (*Mitf*, *Dct*, *Kit*, and *Mlana*), and adipocytes (*Pparγ*, *Adipoq*, *Plin1*, and *Fabp4*) under different induction media. *, compared with ITGA6^neg^ for the top two panels, compared with ITGA6^bright^ for the bottom one, *p < 0.05; **p < 0.01; ***p < 0.001; #, compared with ITGA6^dim^, #*p* < 0.05; ##*p* < 0.01. **(C)** Immunofluorescence staining for tubulin βIII, p75, and PMEL in differentiated ITGA6^bright^ and ITGA6^dim^ cells; scale bar for the left column, 100 μm; for the right four columns, 20 μm.

Neural crest-derived cells have been reported to give rise to glial, melanocytic, and fibroblastic lineages in the skin (Nitzan et al., 2013). To explore the identity and relationship between the three subpopulations, newly isolated ITGA6^neg^, ITGA6^dim^, and ITGA6^bright^ cells from the craniofacial and trunk skin of newborn mice (P0) were cultured and induced to Schwann cells, melanocytes, or adipocytes, respectively. Under Schwann cell induction signals, only ITGA6^bright^ cells showed typical Schwann cell morphology, that is, spindle-shaped cell bodies, long bipolar projections, and highly expressed Schwann cell marker genes, such as *Mbp*, *Mpz*, *p75NTR*, and *Sox10*, as determined by qPCR (Figures 3A, B). In addition to *p75NTR*, primary cultured Schwann cells have also been reported to express neuronal marker tubulin βIII (Jouhilahti et al., 2008; Shen M. et al., 2012). We checked their expression in induced ITGA6^bright^ cells and found a strong immunoreactivity for both of that (Figure 3C). These results suggested that ITGA6^bright^ cells could effectively differentiate into Schwann cells and neurons. Under melanocyte differentiation signals, while most of the cells in the ITGA6^neg^ population died, ITGA6^dim^ cells proliferated, showed typical melanocyte morphology, and melanin granules were visible within the cells, and the cells expressed high levels of melanocyte-specific markers, namely, *Mitf*, *Dct*, *Kit*, and *Mlana* (Figures 3A, B) and stained positive for premelanosome protein (PMEL) (Figure 3C). Given that mouse fibroblasts can effectively differentiate into adipocytes, we next compared the adipogenic potential of the three subpopulations. ITGA6^neg^ cells displayed the strongest adipogenic potential among the three subpopulations, as indicated by Oil Red O staining and qRT-PCR analysis for the expression of *Pparγ*, *Adipoq*, *Plin1*, and *Fabp4* (Figures 3A, B). Combining these results suggested that ITGA6^bright^ and ITGA6^dim^ cells were more likely to differentiate into Schwann cells and melanocytes, respectively, while ITGA6^neg^ cells were more like neural crest-derived fibroblasts.

### 3.4 The transcriptome profiles of ITGA6^bright^ cells revealed their peripheral glial lineage

To further characterize the neural crest-derived ITGA6^bright^, ITGA6^dim^, and ITGA6^neg^ cells in the skin, we isolated the three subpopulations and GFP^neg^ ITGA6^neg^ cells, a fibroblast population and served as a control, from the craniofacial and trunk skin of newborn mice and performed RNA-seq in the three biological replicates. Over 43 million clean reads were generated from each sample, and 90% of these were matched to the mouse genome (Supplementary Figure S2A). Pearson’s correlation coefficient was calculated for all the expressed genes. All the biological replicates displayed a high correlation (*r* > 0.94). Meanwhile, ITGA6^bright^ and ITGA6^dim^ cells exhibited only low similarity to the other groups (*r* < 0.90), whereas ITGA6^neg^ and GFP^neg^ ITGA6^neg^ cells were highly correlated (*r* > 0.94) (Supplementary Figure S2B). Principal component analysis and hierarchical clustering showed the same tendency (Supplementary Figures S2C, S2D), i.e., ITGA6^bright^, ITGA6^dim^, and ITGA6^neg^ cells were clustered separately, whereas ITGA6^neg^ and GFP^neg^ ITGA6^neg^ were clustered together, indicative of the distinctive features of these cell subpopulations and the common characteristics of ITGA6^neg^ and GFP^neg^ ITGA6^neg^ cells. Consistent with the FACS analysis, *Itga6* expression at the mRNA level also discriminated the cell subpopulations as the FPKM indicated (Supplementary Figure S2E).

**FIGURE 4:**
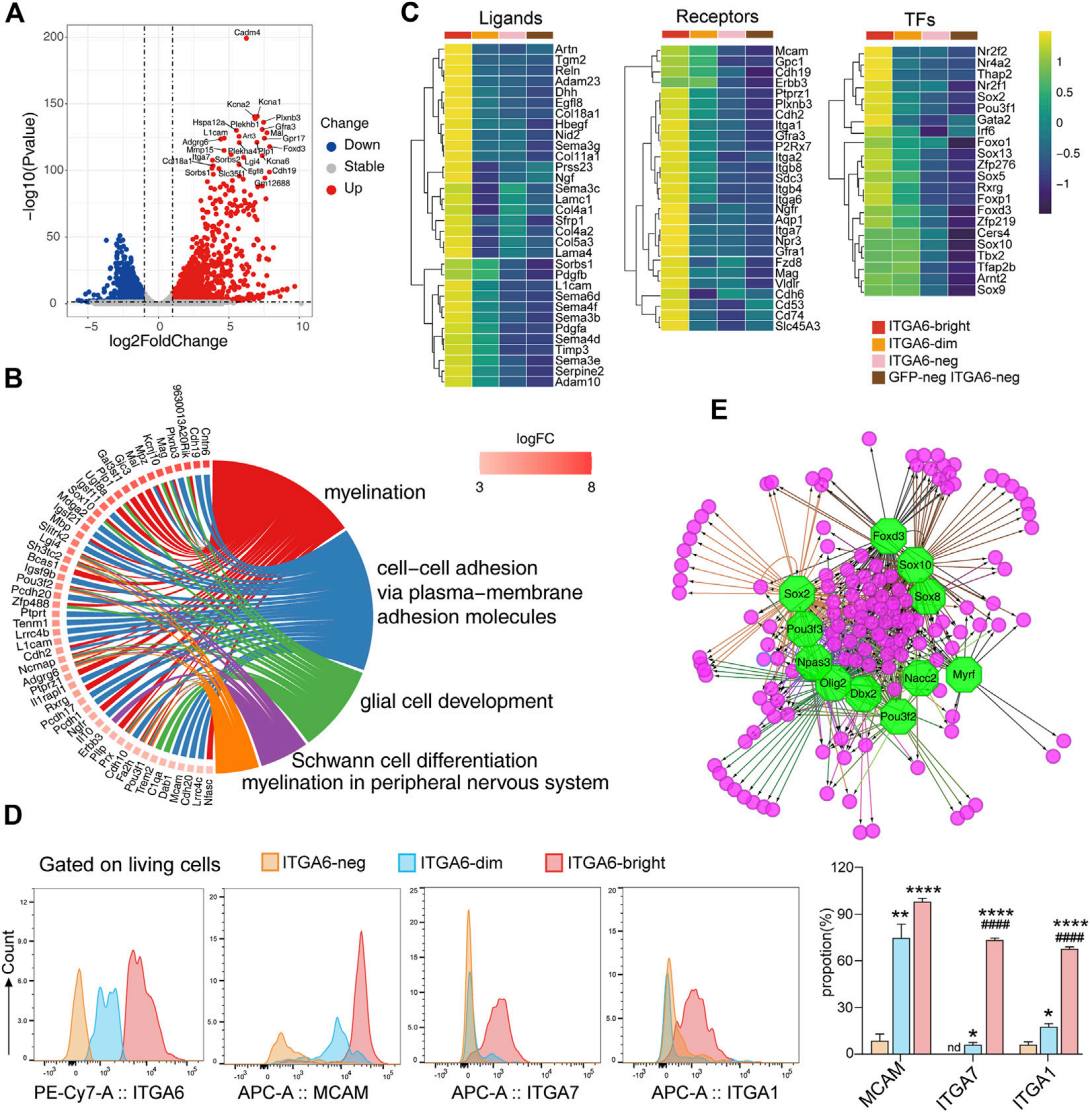
Transcriptomic profiles of ITGA6^bright^ cells reveal their peripheral glial lineage. **(A)** Volcano plot showing the differentially expressed genes between ITGA6^bright^ and GFP^neg^ ITGA6^neg^ cells (*p*-value < 0.05, absolute log2 Fold Change >1). **(B)** GOChord plot of the top GO terms within the biological process (BP) subontology. The upregulated genes in ITGA6^bright^ cells are linked to their assigned pathways by ribbons and ordered according to log2 of the fold-change values (from high to low) represented by a color gradient indicated by the logFC bar. **(C)** Heatmap of selective ligand-, receptor-, and transcription factor (TF)-coding genes highly expressed in ITGA6^bright^ cells. **(D)** Flow cytometry results for the expression of ITGA6, MCAM, ITGA7, and ITGA1 on ITGA6^bright^, ITGA6^dim^, and ITGA6^neg^ cells. *, compared with ITGA6-neg, *p < 0.05; **p < 0.01; ****p < 0.0001; #, compared with ITGA6-dim, #*p* < 0.05; ####*p* < 0.0001. **(E)** Regulatory network analysis showing the predicted TFs (octagonal nodes) among the enriched genes (rounded nodes) in ITGA6^bright^ cells.

Compared with GFP^neg^ ITGA6^neg^ cells, which strongly expressed fibroblast markers such as *Col1A1*, *Col3a1*, *Thy1*(*Cd90*), *Cd34*, and *Twist2*, 2,595 genes were upregulated (adjusted *p*-value < 0.05) in the ITGA6^bright^ subpopulation (Supplementary Figure S2H). Of these, Schwann cell signature genes, such as *Cadm4*, *Mbp*, *Mpz*, *Plp1*, and *Sox10*, and non-myelinating Schwann cell markers, such as Scn7a and Igfbp7, as well as ion channel-related genes, such as *Kcna1* and *Kcna2*, were highly expressed (Figure 4A; Supplementary Figure S2F). GO terms related to Schwann cell differentiation were significantly enriched, including “myelination,” “glial cell development,” and “myelination in the peripheral nervous system” (Figure 4B). We then compared it with a mesenchymal cell population we previously reported (Yang et al., 2020), which was also isolated based on the strong staining of ITGA6 and displayed differentiation potential toward neural crest derivatives. A much higher expression levels of *Mpz*, *Mbp*, *Ptprz1*, and *Plp1* are observed in ITGA6^bright^ cells, whereas mesenchymal genes, such as *Acta2*, *Mest*, *Col1a1*, and *Thy1*, were less frequently detected (Supplementary Figure S3). These results suggest ITGA6^bright^ cells are a distinct cell population and were consistent with the strong differentiation potential of ITGA6^bright^ cells toward a Schwann cell fate observed in the *in vitro* differentiation assay.

Notably, many genes enriched in ITGA6^bright^ cells encode adhesion molecules (Figure 4B), suggesting that interaction between ITGA6^bright^ cells and the microenvironment is important for their development. In addition to *Itgb4* (a subunit binding partner of ITGA6), *Itga1*, *Itga2*, *Itga7*, *Itgb8*, and *Mcam*, and the cadherins Cdh2, Cdh6, and Cdh19, ITGA6^bright^ cells also expressed genes coding for receptors that play a role in neural cell development and maintenance, including PLXNB3, GFRA1, and GFRA3, that preferentially bind SEMA5A, GDNF, and artemin (Figure 4C). Some of these receptors are also important for skin maintenance. For example, the knockout of *Gfra1* was shown to impair the nervous system development and accelerate hair follicle regression (Botchkareva et al., 2000). We also assessed the expression of several adhesion molecules using flow cytometry (Figure 4D). Consistent with the RNA-seq results, we found that ITGA6^bright^ cells expressed high levels of MCAM (CD146), ITGA7, and ITGA1 at the cell surface, suggesting that these molecules could serve as cell-surface markers to identify and isolate Schwann cells in the skin. MCAM could also distinguish ITGA6^bright^, ITGA6^dim^, and ITGA6^neg^ subpopulations.

ITGA6^bright^ cells may also provide important niche factors for both epidermal and dermal cells *via* the secretion of ECM components and signaling molecules such as PDGFA, PDGFB, RELN, and desert hedgehog (DHH) (Figure 4C). Multiple genes coding for ECM components that were enriched in ITGA6^bright^ cells were related to the basement membranes, such as *Col4a1*, *Col4a2*, *Col5a3*, *Col18a1*, *Lama4*, *Lamac1*, and *Nid2*. Mutations in *Reln*, an ECM molecule reported to control cell–cell interactions critical for cell positioning and neuronal migration during brain development (Jossin, 2020), also result in integumentary phenotypes. Dhh, a secreted intercellular signal transducer and lineage tracing marker for peripheral glia (Jaegle et al., 2003), may exert its effects on epidermal stem cell maintenance by regulating the hedgehog activity.

Several genes for neural crest lineage-specific transcription factors were expressed in both ITGA6^bright^ and ITGA6^dim^ cells, including *Sox10*, *Tfap2b*, and *Sox9*, suggestive of a common developmental origin. In contrast, genes encoding transcription factors, such as *Nr2f1*, *Nr2f2*, *Nr4a2*, *Thap2*, *Gata2*, *Irf6*, *Sox2*, *Rxrg*, *Sox5*, *Sox13*, *Foxd3*, and *Foxp1*, were specifically enriched in ITGA6^bright^ cells (Figure 4C). Furthermore, transcription factor prediction results indicated that the genes enriched in ITGA6^bright^ were either individually or jointly regulated by Sox10, Sox2, Foxd3, Pou3f2, Olig2, Dbx2, Myrf, and Nacc2, among other factors, implying that the regulatory networks associated with these transcription factors play roles in Schwann cell development and maintenance (Figure 4E).

### 3.5 The ITGA6^dim^ population was enriched in melanocyte lineage cells

**FIGURE 5:**
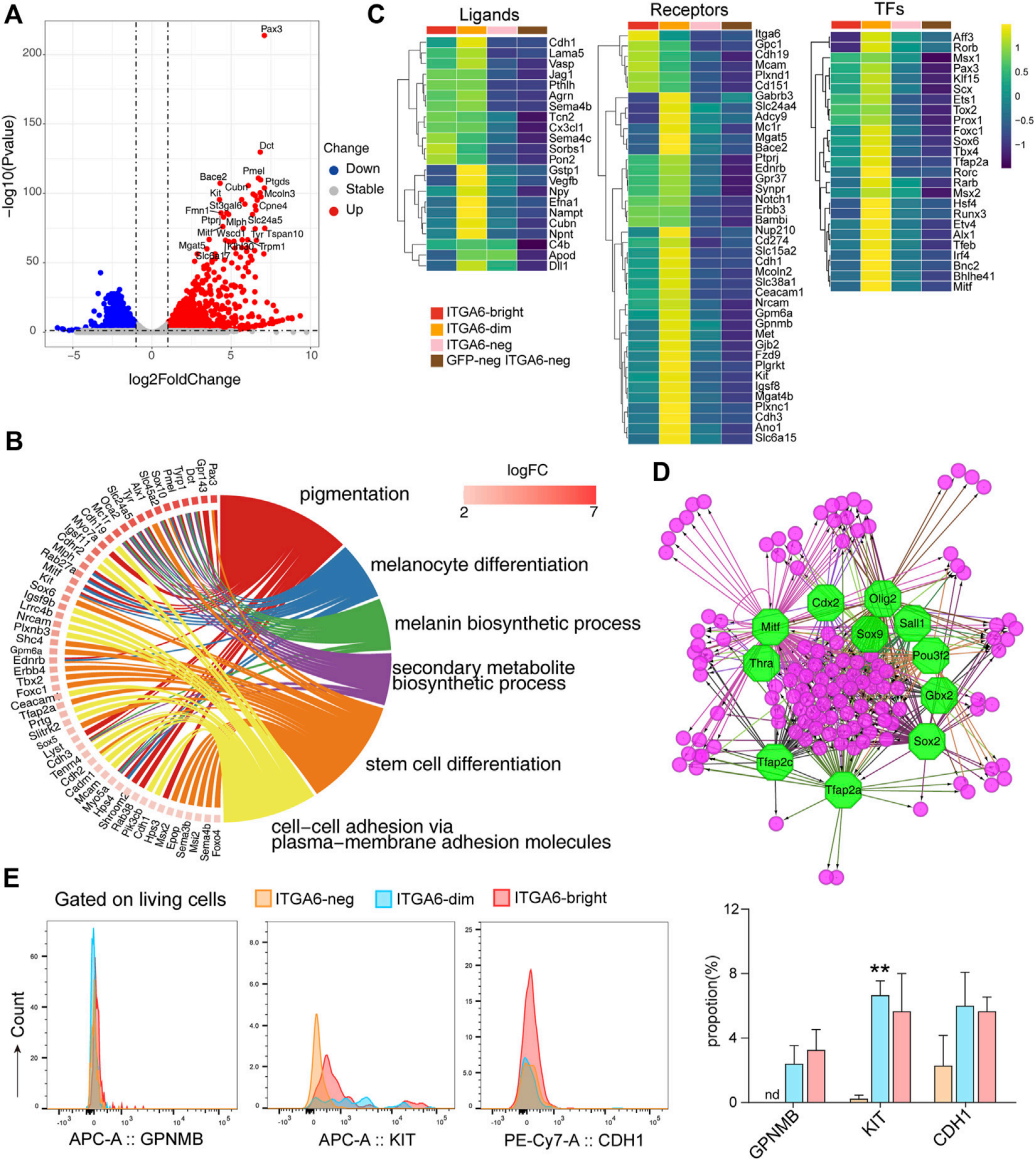
ITGA6^dim^ cells expressing signature genes of the melanocyte lineage. **(A)** Volcano plot showing differentially expressed genes between ITGA6^dim^ and GFP^neg^ ITGA6^neg^ cells. **(B)** GOChord plot displaying the genes specifically expressed in ITGA6^dim^ cells and the top enriched GO biological process terms. **(C)** Heatmap of selective ligand-, receptor-, and transcription factor (TF)-coding genes highly expressed in ITGA6^dim^ cells. **(D)** Regulatory network analysis of the predicted TFs (octagonal nodes) among the genes enriched (round nodes) in ITGA6^dim^ cells. **(E)** Expression of GPNMB, KIT, and CDH1 on ITGA6^bright^, ITGA6^dim^, and ITGA6^neg^ cells as determined using flow cytometry. *, compared with ITGA6^neg^, **p < 0.01.

To uncover the origin and identity of cells in the ITGA6^dim^ subpopulation in the skin, we compared the expression profiles of ITGA6^dim^ and GFP^neg^ ITGA6^neg^ cells. We found that 2,059 genes were upregulated in ITGA6^dim^ cells (Supplementary Figure S2G). Previously described melanocyte development- and melanin metabolism-related genes, such as *Mitf*, *Tyrp1*, *Tyrp2* (*Dct*), *Pmel*, *Kit* (*CD117*), *Mlana*, *Gpnmb*, and *Mcoln3*, were specifically enriched in ITGA6^dim^ cells (Figure 5A). GO terms in biological process associated with “pigmentation,” “melanin biosynthetic process,” and “melanocyte differentiation” were preferentially enriched in ITGA6^dim^ cells (Figure 5B).

Similar to ITGA6^bright^ cells, ITGA6^dim^ cells also expressed high levels of the Notch1, Erbb3, BAMBI, EDNRB, and SYNPR receptors, among others, indicating that they play a role in neural crest cell development (Figure 5C). Compared with ITGA6^bright^ cells, the expression of *Itga6*, *Cdh19*, *Mcam*, *Gpc1*, *Plxnd1*, and *Cd151* was decreased in ITGA6^dim^ cells, suggesting that the resulting downregulation of signals downstream of these molecules may play a role in melanocyte differentiation. ITGA6^dim^ cells uniquely expressed *Kit*, *Met*, *Mc1r*, and *Gjb2. Kit* and *Met* encode receptors for the stem cell factor (SCF) and hepatocyte growth factor (HGF), respectively, which were consistent with previous studies demonstrating the key roles of these molecules in growth, survival, and the migratory behavior of melanocytes (Goding, 2000; McGill et al., 2006). MC1R is primarily located on the surface of melanocytes, where it controls which type of melanin is produced (Wolf Horrell et al., 2016). The activation of MC1R by *α*-melanocyte stimulating hormone (α-MSH) leads to the activation of the cAMP cascade and the expression of genes such as *MITF*, while cellular levels of cAMP are determined by the relative activity of adenylate cyclases (ADCYs) and phosphodiesterases (PDEs). We observed that genes coding for both types of factors, such as *Adcy9*, *Pde1c*, and *Pde8a*, were upregulated in ITGA6^dim^ cells. GJB2, also known as connexin 26 (Cx26), forms gap junction channels, and its mutation has been found to be digenic with other genes mutation in Waardenburg syndrome, which is characterized by varying degrees of deafness and pigmentation (coloring) abnormalities in the eyes, hair, and/or skin (Yan et al., 2011; Kim et al., 2016; Wang P. et al., 2020). ITGA6^dim^ cells also showed the strongest expression of genes encoding the solute carrier family members, including *Slc24a5*, *Slc38a1*, *Slc45a2*, *Slc6a15*, and *Slc6a17* (Figure 5C). *Slc45a2* encodes a putative transporter expressed primarily in pigment cells and controls pH and ionic homeostasis within melanosomes. *S*LC45A*2* mutations and polymorphisms cause oculocutaneous albinism (OCA) and pigmentation variation. To identify specific markers for ITGA6^dim^ cells, we then evaluated the expressions of GPNMB, KIT, and CDH1 (CD324) using flow cytometry. We found that KIT expression was indeed upregulated in ITGA6^dim^ cells (Figure 5E).

ITGA6^dim^ cells uniquely expressed several ligands, including GSTP1, VEGFB, neuropeptide-Y (NPY), EFNA1, and NPNT (Figure 5C), which are known to exert autoregulatory effects and also regulate other cell types in the skin under both physiological and pathological conditions. GSTP1, an isozyme that plays an important role in the detoxification of oxidative byproducts generated during melanin synthesis, has been associated with melanoma susceptibility (Denat et al., 2014). NPY is a 36-amino acid peptide that can induce melanocyte dendricity (Laddha et al., 2014). Given that one of five receptors for NPY is present on immune cells, NPY might play a critical role in the pathogenesis of vitiligo *via* a neuro-immune mechanism involving melanocytes (Farzi et al., 2015). In addition, *via* its immunoregulatory role and mitogenic properties, NPY influences the function of various types of skin cells during the wound-healing process (Ekstrand et al., 2003). ITGA6^dim^ cells also highly expressed Cubilin (CUBN), the endocytic receptor for intrinsic factor-vitamin B12, albumin, and apolipoproteinA-I/HDL (Kozyraki and Cases, 2020). However, its role in melanocytes and other skin cell types remains unknown.

Compared with ITGA6^bright^ cells, the expression of *Sox2*, *Sox5*, *Rxrg*, and *Foxd3* were downregulated in ITGA6^dim^ cells, whereas that of *Pax3*, *Klf15*, *Scx*, *Ets1*, *Foxc1*, *Sox6*, *Tbx4*, and *Tfap2a*, among other genes, was upregulated, which was indicative of the dynamic changes in gene transcription that occur during melanocyte fate determination (Figure 5C). For example, a gradual downregulation of Sox2 in progenitors permits the differentiation of both neural crest- and Schwann cell precursor-derived progenitors into melanocytes (Adameyko et al., 2012). The downregulation of *Foxd3* is recognized as the trigger for melanoblast lineage differentiation. FOXD3 interacts with PAX3 and prevents it from binding to and activating MITF, the master regulator of melanogenesis. The conditional knockout of *Foxd3* in the neural crest results in a glial-to-melanoblast fate switch, whereas its overexpression represses *Mitf* expression and suppresses melanocyte formation (Kos et al., 2001; Nitzan et al., 2013). Here, we observed that besides *Mitf*, other transcription factor-encoding genes, such as *Rarb*, *Hsf4*, *Runx3*, *Etv4*, *Alx1*, *Tfeb*, *Bnc2*, and *Bhlhe41*, were also uniquely expressed in ITGA6^dim^ cells (Figure 5C), suggesting that they exert regulatory roles in melanogenesis. Transcription factor analysis of ITGA6^dim^ signature genes further indicated that regulatory networks mediated by SOX2, TFAP2A, TFAP2C, MITF, OLIG2, POU3F2, SALL1, GBX2, THRA, and CDX2 are involved in melanocyte development (Figure 5D). Among these, CDX2 is a member of Cdx proteins, which has been proven to play a key early role in the trunk neural crest gene regulation networks, especially for melanocyte development by directly controlling the expression of Pax3, Msx1, and Foxd3 (Sanchez-Ferras et al., 2016).

### 3.6 ITGA6^neg^ and GFP^neg^ ITGA6^neg^ cells represent fibroblasts of different origins

**FIGURE 6:**
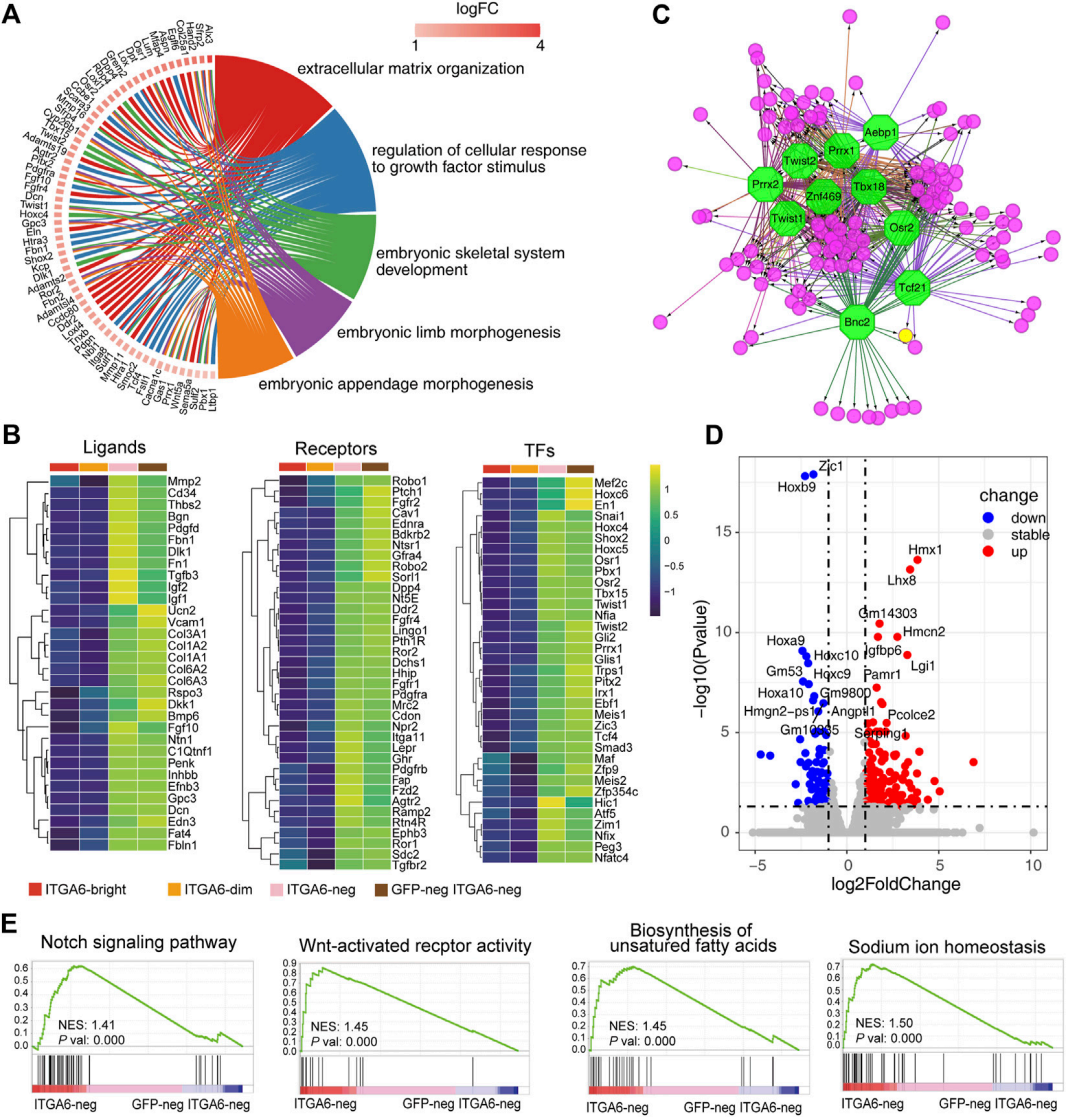
ITGA6^neg^ and GFP^neg^ ITGA6^neg^ marked fibroblasts of different origins. **(A)** GOChord plot of the highly expressed genes in ITGA6^neg^ and GFP^neg^ ITGA6^neg^ cells and their corresponding GO terms. **(B)** Heatmap of selective ligand, receptor-, and transcription factor (TF)-coding genes highly expressed in ITGA6^neg^ and GFP^neg^ ITGA6^neg^ cells. **(C)** Regulatory network analysis of the predicted TFs (octagonal nodes) among the enriched genes (rounded nodes) in ITGA6^neg^ and GFP^neg^ ITGA6^neg^ cells. **(D)** Volcano plot showing the differentially expressed genes (*p*-value < 0.01, absolute log2 Fold Change >1) between ITGA6^neg^ and GFP^neg^ ITGA6^neg^ cells. **(E)** Gene set enrichment analysis plots of the highly expressed genes in ITGA6^neg^ cells relative to GFP^neg^ ITGA6^neg^ cells.

As both Pearson correlation and cluster analysis indicated that the ITGA6^neg^ and GFP^neg^ ITGA6^neg^ cell subpopulations were highly similar, we next analyzed 859 genes that were highly expressed in both groups (Supplementary Figure S2G). Gene enrichment analysis showed that these genes were mainly involved in mesenchymal development, as evidenced by the significant enrichment of GO terms such as “extracellular matrix organization,” “embryonic skeletal system development,” “embryonic limb morphogenesis,” and “embryonic appendage morphogenesis” (Figure 6A). Additionally, mesenchymal development-related genes were highly expressed in both ITGA6^neg^ and GFP^neg^ ITGA6^neg^ cells, including those coding for collagens and their assembly or degradation-related factors, such as *Col1a2*, *Col3a1*, *Loxl1*, *Loxl4*, *Loxl2*, *Adamts2*, *Ctsk*, *Adamts2*, *Lum*, *Ptgfr*, *Pid1*, *Cyp26b1*, *Ifitm1*, *Ank2*, and *Hecw2*, suggesting that both cell subpopulations shared fibroblastic identity (Figure 6B).

ITGA6^neg^ and GFP^neg^ ITGA6^neg^ cells strongly expressed genes encoding classical surface receptors of stromal cells such as *Fgfr1*, *Fgfr2*, *Fgfr4*, *Tgfbr2*, *Pdgfra*, *Pdgfrb*, *Dpp4*, *Fap*, *Ddr2*, and *Mrc2* (Figure 6B), which are associated with the GO biological process of “regulation of cellular response to growth factor stimulus.” *Ddr2* and *Mrc2* encode collagen metabolism-related receptors. The deficiency of DDR2 leads to delayed wound healing (Cario, 2018). MRC2 may contribute to cellular uptake, remodeling, and degradation of extracellular collagen matrices (Lopez-Guisa et al., 2012). Furthermore, the genes encoding the Hedgehog receptor PTCH1, the Wnt receptor FZD2, and the Wnt modulators ROR1 and ROR2 were also highly expressed in the two cell populations. Interestingly, we found a distinct set of receptors for neuro-endocrine factors that were enriched in ITGA6^neg^ and GFP^neg^ ITGA6^neg^ cells, including neurotensin receptor 1 (*Ntsr1*), natriuretic peptide receptor 2 (*Npr2*), *Lepr*, *Ramp2*, *Pth1r*, and *Ghr*, suggesting that fibroblast development and homeostasis may involve neuroendocrine regulation.

In addition to ECM molecules such as collagens, fibronectin 1, thrombospondin, and decorin, ITGA6^neg^ and GFP^neg^ ITGA6^neg^ cells also strongly expressed a series of signaling-related genes, including *Rspo3*, *Dlk1*, *Dkk1*, *Efnb3*, *Edn3*, *Fgf10*, *Pdgfd*, *Penk*, *Bmp6*, *Inhbb*, *Igf1*, *Igf2*, and *Ntn1* (Figure 6B), which are important for skin development and maintenance. For instance, RSPO3 functions as an activator of the canonical Wnt signaling pathway by acting as a ligand for LGR4–6 receptors, which play key regulatory roles in angiogenesis (Kazanskaya et al., 2008). FGF10 is an important mediator of epithelial–mesenchymal interaction and participates in hair follicle development (Suzuki et al., 2000). Netrin 1 (Ntn1) is a laminin-related secreted protein involved in axon guidance and cell migration during development (Xu et al., 2014). The receptors for some of the aforementioned ligands are expressed on other types of cells in the skin. For instance, the EDN3 receptors, EDNRB and Notch1, were mainly expressed on ITGA6^bright^ and ITGA6^dim^ cells, respectively, while the PENK receptor is expressed on keratinocytes (Nagui et al., 2016), suggesting that ITGA6^neg^ and GFP^neg^ ITGA6^neg^ cells may constitute a specific niche supporting the development or maintenance of other skin cells.

We further revealed a series of genes encoding skin fibroblast-related transcription factors, including *En1*, *Snai1*, *Twist1*, *Twist2*, *Meis1*, *Meis2*, *Pbx1*, *Hoxc4*, *Hoxc5*, and *Hoxc6* (Figure 6B). This is consistent with a previous study reporting that Meis and Pbx proteins form a complex and bind to DNA through respective Meis and Pbx consensus binding sites and thereby regulate the transcription of Hox genes (Garcia-Cuellar et al., 2015). Other transcription factors may also regulate the development of fibroblasts, including SHOX2, TBX15, PEG3, ZIM1, HIC1, TRPS1, PITX2, GLI1, GLI2, NFIA, and NFIX. Of these, TBX15 has been reported to be involved in the control of mesoderm specification. The inactivation of the *Tbx15* gene in mice and mutations of TBX15 in humans results in severe skeletal malformations (Singh et al., 2005; Lausch et al., 2008). *Trps1* was reported to play a role in regulating genes that control the growth of bone and other skeletal tissues, and the aberrant expression of the human homolog of TRPS1 leads to trichorhinophalangeal syndrome (Momeni et al., 2000). *Hic1* is broadly expressed within embryonic mesenchyme, and mice deficient for *Hic1* die prenatally due to severe developmental defects in craniofacial structures (Ray and Chang, 2020). *Pitx2* mediates FGF10 expression in gut mesenchymal cells in response to FGF9 signals, thus playing a role in epithelial structure formation (Al Alam et al., 2012). The human homolog of *Pitx2* was identified as the gene mutated in Axenfeld–Rieger Syndrome type I, in which the development of the teeth, eyes, and umbilicus is affected (Tumer and Bach-Holm, 2009). *Gli1* and *Gli2* are mediators of Sonic hedgehog (Shh) signaling and were shown to be important for the mesenchymal cell cluster-mediated induction of epithelial folding to initiate villus emergence (Coquenlorge et al., 2019). Moreover, using the transcription factor prediction analysis, we also identified the 10 most enriched transcription factors involved in the regulation of the signature genes of ITGA6^neg^ and GFP^neg^ ITGA6^neg^ cells, namely, TBX18, ZNF469, TWIST1, TWIST2, PRRX1, PRRX2, AEBP1, OSR2, BNC2, and TCF21 (Figure 6C).

As ITGA6^neg^ and GFP^neg^ ITGA6^neg^ cells were derived from neural crest and mesoderm, respectively, we wondered whether there were some characteristic differences between the two groups of cells. We compared the gene expression profiles of both the cell groups and identified 373 differentially expressed genes, 241 of which were upregulated and 132 were downregulated (Figure 6D and Supplementary Figure S2H). As shown in the volcano plot in Figure 6D, the expression of *Hmx1*, *Lhx8*, *Gm14303*, *Igfbp6*, *Hmcn2*, *Lgi1*, *Pamr1*, *Angptl1*, *Pcolce2*, and *Serping1* was markedly increased in ITGA6^neg^ cells compared with that in GFP^neg^ ITGA6^neg^ cells. This suggested that differential regulatory mechanisms were involved in the development of the two cell subpopulations. Meanwhile, the most significantly upregulated genes in GFP^neg^ ITGA6^neg^ cells were *Zic1* and homeobox genes, such as *Hoxb9*, *Hoxa9*, *Hoxc10*, *Hoxc9*, and *Hoxa10* (Figure 6D), suggesting that these transcription factors participate in the differentiation of fibroblasts derived from the dermomyotome or lateral plate mesoderm. Gene set enrichment analysis (GSEA) showed that genes related to the Notch and Wnt signaling pathways, the biosynthesis of unsaturated fatty acids, and sodium ion homeostasis were enriched in ITGA6^neg^ cells relative to GFP^neg^ ITGA6^neg^ cells (Figure 6E), suggesting that these pathways are involved in regulating the differentiation of neural crest-derived fibroblasts.

Taken together, these results demonstrated that while ITGA6^neg^ and GFP^neg^ ITGA6^neg^ cells showed highly similar expression of fibroblast-specific functional genes, transcription factors, ligands, and receptors, specific regulatory elements exist in these cells that reflect their different developmental origins.

## 4 Discussion

In this study, using a combination of neural crest lineage tracing and flow cytometry, we found that the expression of ITGA6 in neural crest and its derivatives was developmentally regulated. Based on the expression of ITGA6, Wnt1-Cre lineage neural crest derivatives in the skin could be categorized into ITGA6^bright^, ITGA6^dim^, and ITGA6^neg^ subpopulations, which were found to be Schwann cells, melanocytes, and fibroblasts, respectively, as determined using an *in vitro* differentiation assay and transcriptomic analysis. We further assessed the signature genes, ligands, receptors, and transcription factors enriched in the cell subpopulations and predicted the transcription factors responsible for the differences in gene expression. Additionally, we identified the molecules that are specifically expressed in neural crest and mesoderm-derived fibroblast, which may play an essential role in the development of fibroblast with different origins. These results provide insights into the regulatory landscape of neural crest cell development in the skin.

Our results showed that ITGA6 could distinguish different neural crest derivatives in the skin. The single-cell RNA-seq data displayed that *Itga6* could discriminate different neural crest clusters due to their hierarchical expressions. The differential expression levels of ITGA6 are also observed in derivatives of neural crest cells after they migrate to and settle in the skin, where Wnt1-Cre lineage-derived ITGA6^bright^, ITGA6^dim^, and ITGA6^neg^ subpopulations display complete gene profiles of Schwan cells, melanocytes, and fibroblasts, respectively. These results indicated that ITGA6 may serve as a specific surface marker of neural crest derivatives in the skin, which is useful for identifying and enriching these cell populations, as well as for further understanding the regulatory mechanisms involved in neural crest differentiation. Additionally, given that integrins transmit both “inside-out” and “outside-in” signals (Streuli, 2009), the differential expression of ITGA6 may serve as an important regulatory mechanism in neural crest development through sensing and interacting with environmental cues, as previously proposed. For example, when cultured on high concentrations of laminin, cranial neural crest cells migrate at approximately twice the rate of trunk neural crest cells due to exhibiting the lower surface expression levels of ITGA6. Overexpressing ITGA6 in cranial neural crest cells cultured on high laminin densities can significantly slow their migration rate (Strachan and Condic, 2003). In this way, it is worthy to study whether and how ITGA6 exerts effects on the development of neural crest cell after settling into the skin tissue.

ITGA6 may also be a marker for the early development of cells or cells in an early differentiation stage beyond the neural crest cells. To date, ITGA6 was commonly found in more than 30 different populations of stem cells, including some cancer stem cells (Krebsbach and Villa-Diaz, 2017). It was also reported that the differential expression of ITGA6 discriminates hematopoietic stem cells (HSCs) and HSC-independent progenitors (Xu et al., 2019). In our previous works, we found ITGA6 labels different subpopulations of skin mesenchymal cells, which displayed neural crest-like cell properties, and account for a higher percentage in fetal skin than that in adults (Yang et al., 2020). Interestingly, ITGA6-high mesenchymal cells also express high levels of ITGA7, ITGA1, and MCAM, further suggesting these adhesion molecules may work synergistically. Considering this, we speculate that Wnt1-Cre lineage-derived ITGA6^bright^ cells isolated from P0 mice skin are not a homogenous Schwann cell population and may contain Schwann cell precursors (SCPs), which give rise to peripheral neurons, Schwann cells, melanocytes, and neuroendocrine and endoneurial fibroblasts (Kastriti et al., 2022). This is supported by our *in vitro* differentiation assay showing that ITGA6^bright^ cells could also be induced to neurons, melanocytes, and adipocytes, and by transcriptome analysis showing the high-level expressions of both myelinating (*Mpz* and *Mbp*) and non-myelinating Schwann cells (*Scn7a* and *Igfbp7*).

An in-depth analysis of the ligands and receptors expressed by different neural crest-derived subpopulations revealed the interaction between cells of different developmental origins in the skin, as well as the underlying regulatory network. The genes *Pdgfa* and *Pdgfb*, encoding ligands for PDGFRA and PDGFRB, which are present on fibroblasts and perivascular cells (Wang Y. et al., 2020), respectively, were strongly upregulated in ITGA6^bright^ cells (Schwann cells), suggestive of their important role in fibroblast and vascular development. Basal membrane components, such as COL4A1, COL4A2, COL5A3, COL18A1, LAMA4, LAMAC1, and NID2, and type IV collagens secreted by ITGA6^bright^ cells likely contribute to epithelial–mesenchymal interactions during skin/hair follicle development (Hardy and Goldberg, 1983). Different from a previous report showing that NPY was mainly secreted by sympathetic postganglionic nerve fibers (Magnussen et al., 2015), we found that ITGA6^dim^ cells (melanocytes) strongly express NPY. Y1, one of the five receptors for NPY, is present on B cells, T cells, dendritic cells, and macrophages, while NPY has been reported to play an important role in the induction of immune responses by acting on a variety of immune cells (Wheway et al., 2005). These observations suggest that melanocytes are involved in skin immune regulation. Nephronectin (NPNT), which was specifically expressed in ITGA6^dim^ cells, is a member of the epidermal growth factor (EGF) repeat superfamily of proteins. It has been reported to be a smooth muscle cell niche factor, inducing α8 integrin-positive mesenchymal cells to upregulate smooth muscle markers (Fujiwara et al., 2011). Mice with NPNT deficiency have fewer arrector pili muscles in the skin. While the mutation of EDNRB, a receptor for EDN3, led to severe defects in melanocyte numbers in mice (Takeo et al., 2016), we found EDN3 was mainly secreted by fibroblasts, suggesting the importance of fibroblast signals to melanocytes. ITGA6^neg^ cells also strongly expressed the regulators of skin appendage development, such as Wnt ligands RSPO3, DKK1, and FGF10 (Ito et al., 2007). These findings indicated that neural crest derivatives provide specific niche factors that regulate the development and/or maintenance of other skin cells. The molecules identified here will offer a new perspective on understanding skin development and skin disorders.

Fibroblasts have long been known to be derived from mesoderm and neural crest. However, the differences in their molecular signatures are yet to be fully elucidated. Both GFP^neg^ ITGA6^neg^ and ITGA6^neg^ cells expressed high levels of *Pdgfra*, a marker for multiple mesenchymal lineages; ECM molecules such as collagens and fibronectin; and ECM remodeling enzymes such as lysyl oxidase, matrix metalloproteinases (MMPs), and MMP inhibitors, which are fibroblastic features. Among the most significantly differentially expressed genes between the two groups of cells, those coding for transcription factors HMX1 and LHX8 were specifically expressed in ITGA6^neg^ cells. HMX1 and LHX8 have been reported to regulate the development of craniofacial mesenchymal lineages such as bone and cartilage. For example, HMX1 was found to be predominantly expressed in the developing craniofacial mesenchyme, retina, and sensory nervous system (Yoshiura et al., 1998). Mutations in *HMX1* (human *NKX5-3*) alleles cause ear deformities in multiple species, including the “misplaced ears” phenotype in mice (Munroe et al., 2009), the dumbo (dmbo) phenotype of “fancy” rats (Kuramoto et al., 2010), and oculoauricular syndrome in humans (Kuramoto et al., 2010; Si et al., 2020). LHX8, a highly conserved transcription factor of the LIM-homeobox family, is abundantly expressed in specific periods of development of multiple mesenchymal lineages in craniofacial tissues, including bone and dental mesenchyme at the bud stage (E12.5) in the mouse (Kim and Choung, 2020), and plays a crucial role in cell fate regulation. Thus, HMX1 and LHX8 are required for the development of neural crest-derived mesenchyme, including bone, cartilage, tooth, and fibroblast. However, their clear roles in fibroblast differentiation need further investigation. Compared with the fibroblasts derived from the neural crest, *Zic1* and homeobox (HOX) transcription factors were enriched in mesoderm-derived fibroblasts. *Zic1* encodes a member of a conserved family of zinc-finger proteins and has been reported to activate endogenous Myf5 expression in 10T1/2 cells and presomitic mesoderm explants (Pan et al., 2011). Zic1 also interacts cooperatively with Gli proteins to potentiate the transactivation of Gli-dependent Myf5 epaxial somite-specific (ES) enhancer activity in 3T3 cells. Given the close relationship between *Zic1* and *Myf5* expression, we speculate that Zic1 may be specific for somite-derived fibroblasts. However, the specific regulatory role of HMX1, LHX8, and Zic1 in the development of fibroblasts of different origins needs further investigation.

## Data Availability

The datasets presented in this study can be found in online repositories. The names of the repository/repositories and accession number(s) can be found in the article/Supplementary Material.
