# Supplementary figures and images for: Developmentally regulated expression of integrin alpha-6 distinguishes neural crest derivatives in the skin
Source: Front Cell Dev Biol. 2023 May 15;11:1140554. doi: 10.3389/fcell.2023.1140554 (PMC10225710; doi:10.3389/fcell.2023.1140554)

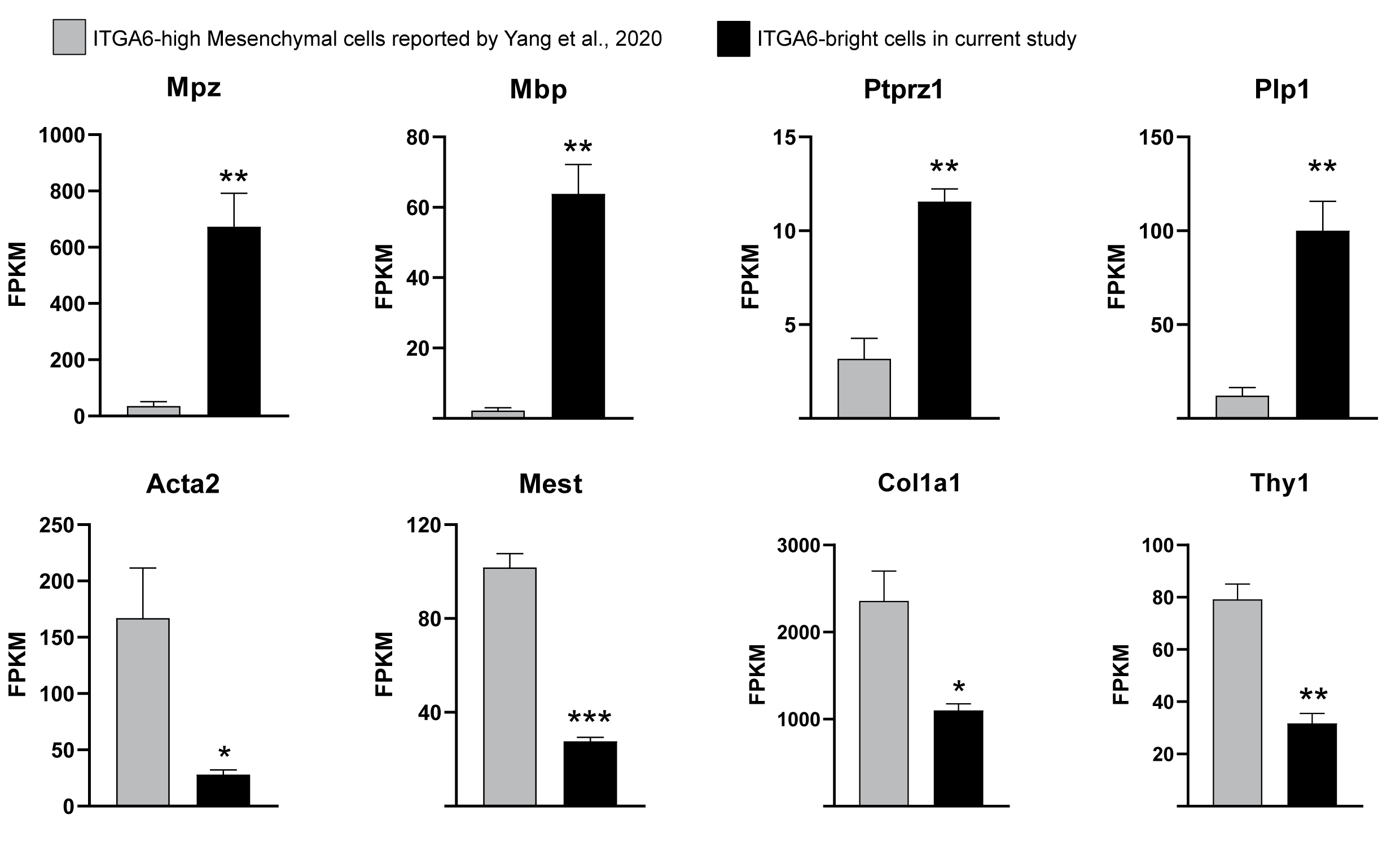

Supplement: Supplementary file 1 [file Image3.TIF]

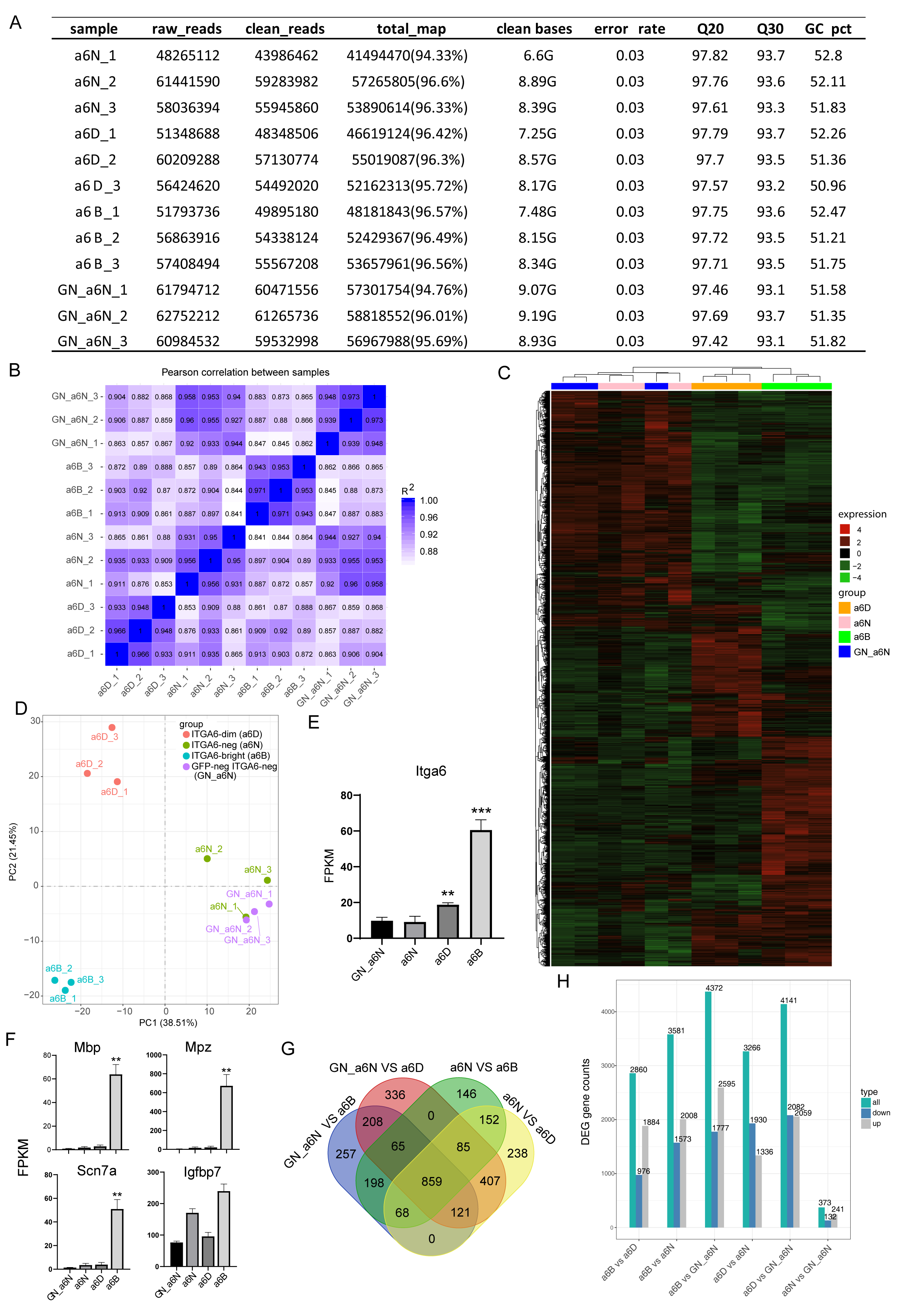

Supplement: Supplementary file 2 [file Image2.TIF]

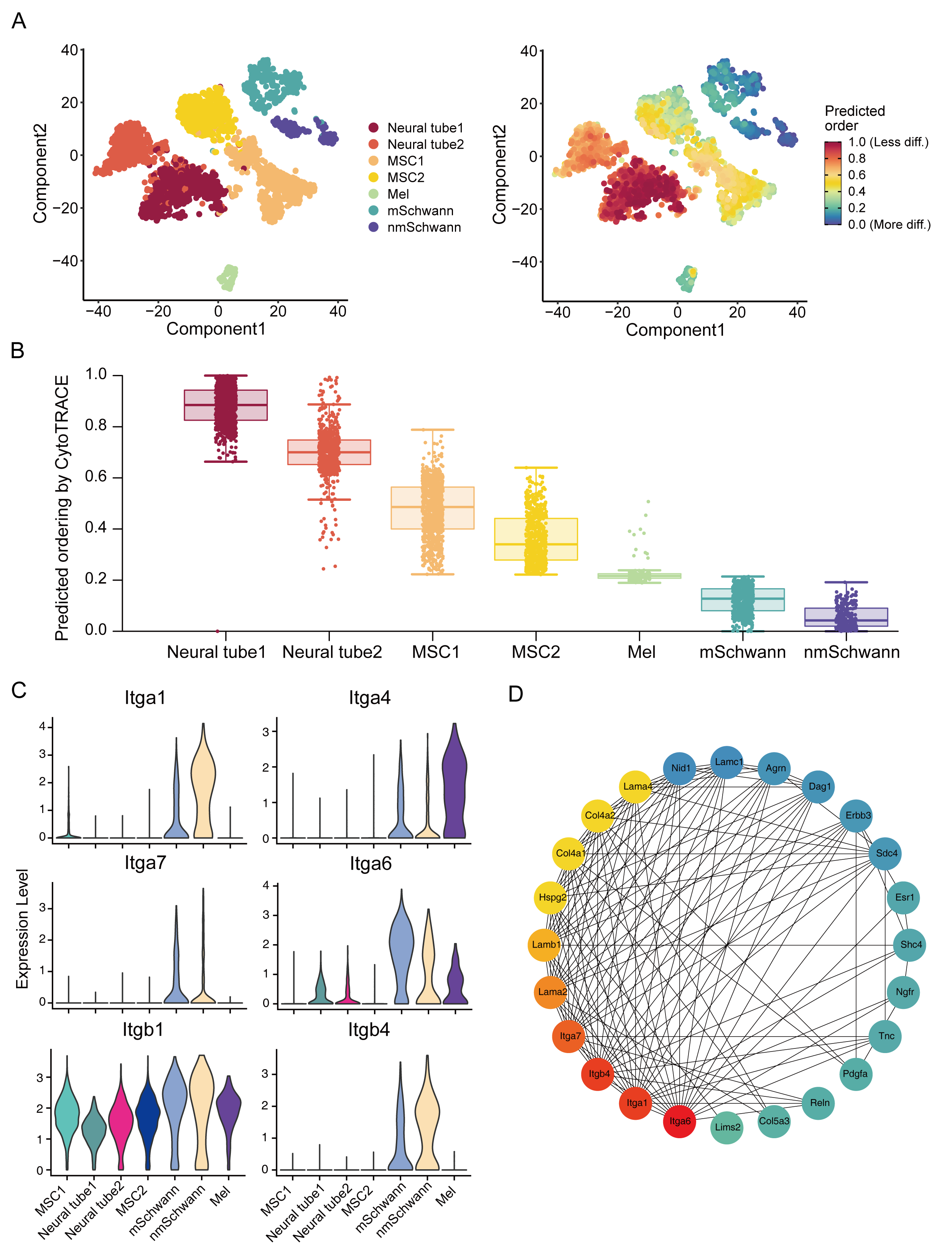

Supplement: Supplementary file 3 [file Image1.TIF]
