# Supplementary material for: Developmentally regulated expression of integrin alpha-6 distinguishes neural crest derivatives in the skin
Source: Front Cell Dev Biol. 2023 May 15;11:1140554. doi: 10.3389/fcell.2023.1140554 (PMC10225710; doi:10.3389/fcell.2023.1140554)
Supplement: Supplementary file 4 [file DataSheet1.PDF]

Supplementary Material

Developmentally Regulated Expression of Integrin Alpha-6 Distinguishes Neural Crest Derivatives in the Skin

Shize Ma, Xiu Li, Rui Cao, Guoqin Zhan, Xin Fu, Ran Xiao, Zhigang Yang*,

***Correspondence:** Zhigang Yang: yangzg@psh.pumc.edu.cn

Supplementary Figures

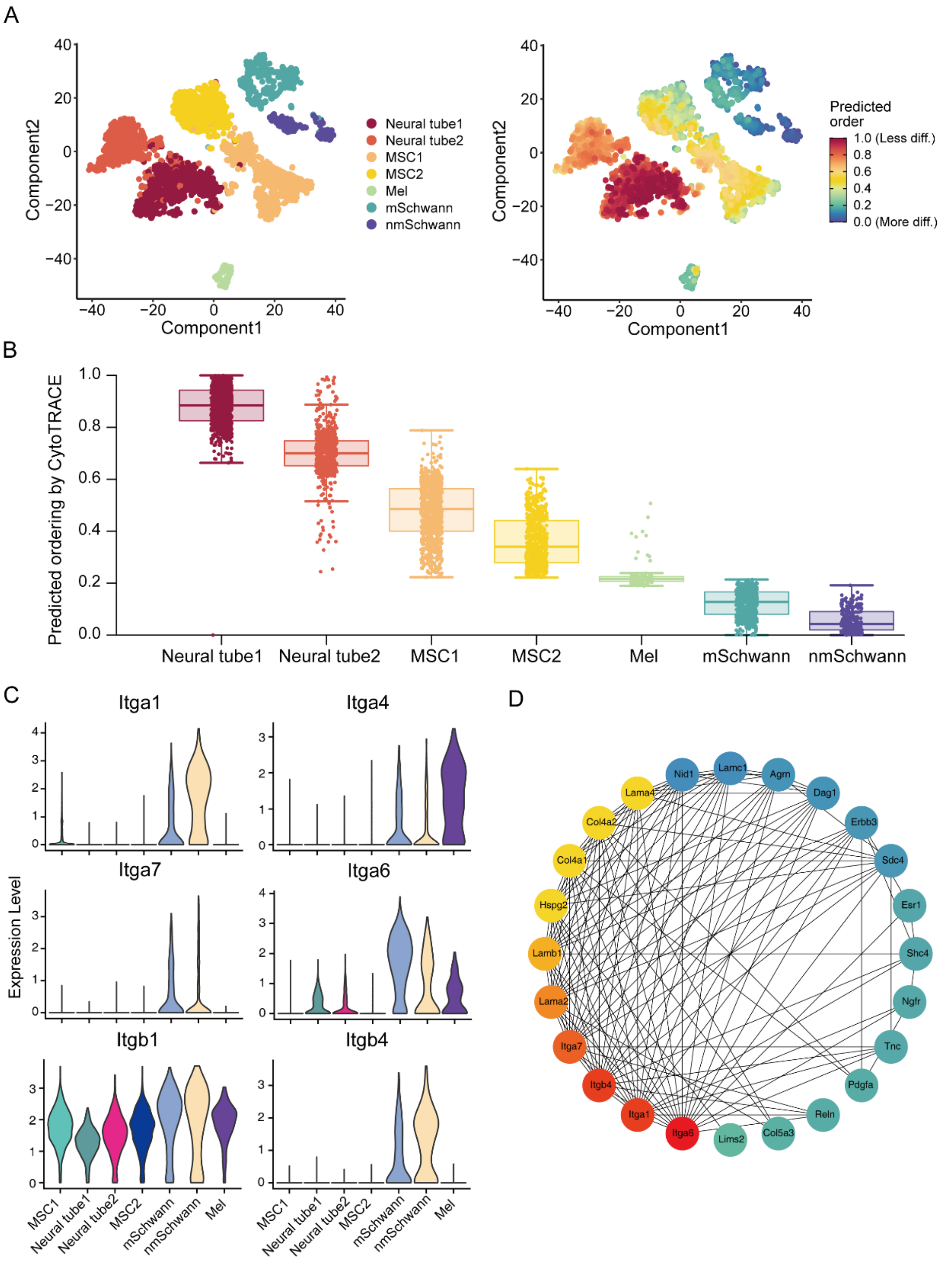

Supplement Figure 1. CytoTRACE analysis of cell state and expressions of Integrins among cell clusters.

A, CytoTRACE analysis to predict the differentiation status of the seven cell clusters. In the architecture, highly differentiated cells have low transcriptional diversity and low CytoTRACE scores, while less differentiated cells have high transcriptional diversity and high scores. B, Boxplot showing the progressively decreasing differentiation ordering predicted by CytoTRACE. C, Violin plots showing the expression levels of integrins that dynamically changed during pseudotime analysis. D, PPI network of ECM-receptor interaction related genes. The colors ranging from red to yellow indicate the ten most significant hub genes with degree measurement from high to low among these molecules.

A

sample	raw_reads	clean_reads	total_map	clean bases	error rate	Q20	Q30	GC pct
a6N_1	48265112	43986462	41494470(94.33%)	6.6G	0.03	97.82	93.7	52.8
a6N_2	61441590	59283982	57265805(96.6%)	8.89G	0.03	97.76	93.6	52.11
a6N_3	58036394	55945860	53890614(96.33%)	8.39G	0.03	97.61	93.3	51.83
a6D_1	51348688	48348506	46619124(96.42%)	7.25G	0.03	97.79	93.7	52.26
a6D_2	60209288	57130774	55019087(96.3%)	8.57G	0.03	97.7	93.5	51.36
a6 D_3	56424620	54492020	52162313(95.72%)	8.17G	0.03	97.57	93.2	50.96
a6 B_1	51793736	49895180	48181843(96.57%)	7.48G	0.03	97.75	93.6	52.47
a6 B_2	56863916	54338124	52429367(96.49%)	8.15G	0.03	97.72	93.5	51.21
a6 B_3	57408494	55567208	53657961(96.56%)	8.34G	0.03	97.71	93.5	51.75
GN_a6N_1	61794712	60471556	57301754(94.76%)	9.07G	0.03	97.46	93.1	51.58
GN_a6N_2	62752212	61265736	58818552(96.01%)	9.19G	0.03	97.69	93.7	51.35
GN_a6N_3	60984532	59532998	56967988(95.69%)	8.93G	0.03	97.42	93.1	51.82

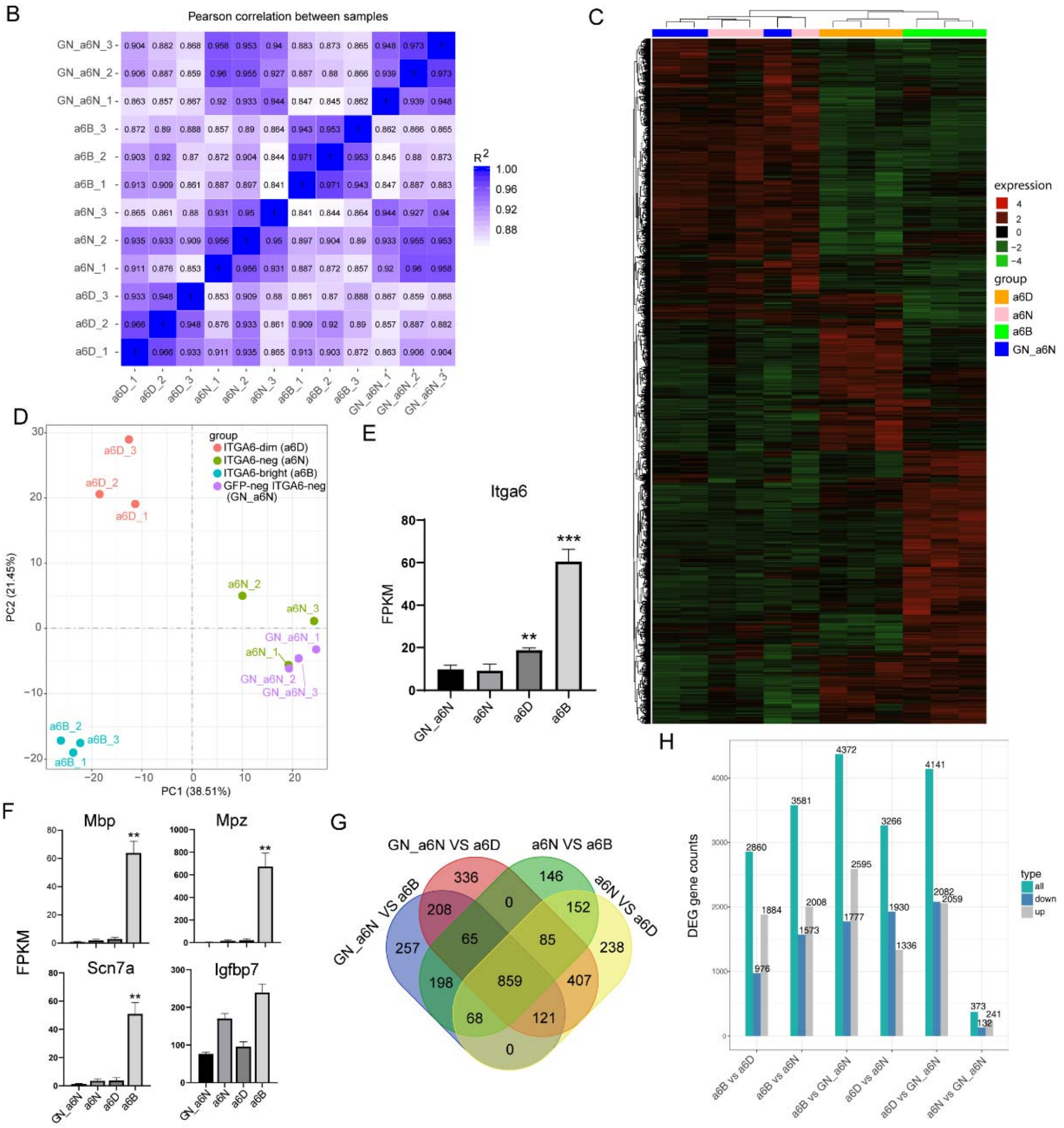

Supplement Figure 2. Overview of RNA-seq data.

A, Summary of the sequence quality, reads number and mapping results for transcriptomic analysis of ITGA6^{bright}, ITGA6^{dim}, ITGA6^{neg}, and GFP^{neg}ITGA6^{neg} cells subpopulations. B, Heatmap of Pearson's correlation coefficient for gene expression profiles of the 4 subpopulations. C&D, Hierarchical clustering and PCA

analysis showing the distinctive features of these subpopulations. E, FPKM values of *Itga6* in different cell subpopulations; *, compared with a6N, **p < 0.01; ***p < 0.001. F, FPKM values of *Mbp*, *Mpz*, *Scn7a*, *Igfbp7* in different cell subpopulations; *, compared with a6N, **p < 0.01. G, Venn diagram showing the numbers of common DEGs among the four different comparison groups. H, Bar graphs showing the numbers of DEGs between different groups of cells.

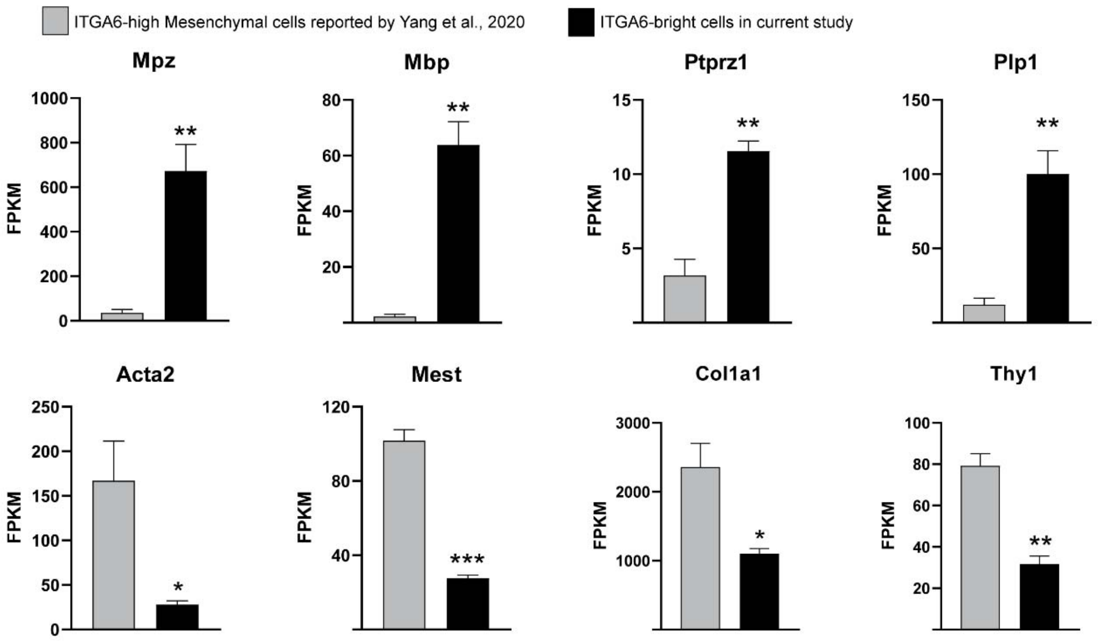

Supplement Figure 3. Differential gene expressions between ITGA6-high mesenchymal cells (Yang et al., 2020) and Wnt1-lineage derived ITGA6-bright cells.
